# Immunosenescence and Immunotherapy in Elderly Acute Myeloid Leukemia Patients: Time for a Biology-Driven Approach

**DOI:** 10.3390/cancers10070211

**Published:** 2018-06-22

**Authors:** Alessandro Isidori, Federica Loscocco, Marilena Ciciarello, Giulia Corradi, Mariangela Lecciso, Darina Ocadlikova, Sarah Parisi, Valentina Salvestrini, Sergio Amadori, Giuseppe Visani, Antonio Curti

**Affiliations:** 1 Haematology and Stem Cell Transplant Center, AORMN Marche Nord Hospital, Via Lombroso, 61100 Pesaro, Italy; federica.loscocco@gmail.com (F.L.); pesarohematology@yahoo.it (G.V.); 2 Department of Experimental, Diagnostic and Specialty Medicine, Institute of Hematology “L. and A. Seràgnoli”, University of Bologna, Via Massarenti, 9, 40138 Bologna, Italy; marilenaci@hotmail.com (M.C.); giulia.corradi2@unibo.it (G.C.); mariangela.lecciso@gmail.com (M.L.); darina.ocadlikova@unibo.it (D.O.); sarah.parisi@alice.it (S.P.); salvestrinivalentina@libero.it (V.S.); 3 GIMEMA Foundation, 00187 Rome, Italy; sergioamadori1946@gmail.com

**Keywords:** acute myeloid leukemia, tumor immunity, immunotherapy, immunosenescence, new drugs, cell therapy

## Abstract

Acute myeloid leukemia (AML) is a disease, which mainly affects the elderly population. Unfortunately, the prognosis of patients aged >65 years is dismal, with 1-year overall survival approaching 10% with conventional therapies. The hypothesis of harnessing the immune system against cancer, including leukemia, has been postulated for a long time, and several clinical attempts have been made in this field. In the last years, we increased our knowledge about the interplay between AML and immune cells, but no major improvement has been translated, up to now, from bench to bedside. However, the outstanding results coming from the modern immuno-oncology trials with new drugs have granted a new interest for immunotherapy in AML. Accordingly, the elderly population represents an ideal target, given the low percentage of patients eligible for allogeneic stem cell transplant. With that in mind, in the era of immunotherapy, we consider immunosenescence as the optimal background to start investigating a biology-driven approach to AML therapy in the elderly. By taking into account the physiological age-related changes of immune response, more personalized and tailored use of the new drugs and strategies harnessing the immune system against AML, has the potential to increase their efficacy and impact on clinical outcomes.

## 1. Introduction

Acute myeloid leukemia (AML) is a clonal disorder, sprouting from a rare population of leukemic stem cells [1]. In the last years, major strides have been made in the understanding of AML biology, which greatly impacted on diagnosis and prognostication, resulting in a new risk-stratification of AML [2]. Although in some cases, such as FLT3-mutated AMLs, these biological achievements have translated in effective new drugs [3], the therapeutic relevance of these advances is still elusive for the majority of AML patients. Consequently, the core management of AML still relies on aggressive chemotherapy, followed by allogeneic stem cell transplantation (SCT) [4]. If such an approach has the potential to cure fit and young patients, the question of effective treatment of unfit and elderly patients is still open and unsolved [5]. Indeed, the poor clinical outcome in this patient population is due to an increase in unfavorable biological features and in the presence of comorbidities, which limits, if not excluded, the possibility of undergoing SCT as a consolidation strategy, whenever complete remission (CR) is achieved. In this scenario, the exploitation of immunological therapies may allow to bypass drug-resistance, thus resulting in improved clinical outcomes, especially in elderly patients. 

Little doubt that the immune system is capable to counteract the development and growth of AML cells exists [6,7]. During the breakdown in cellular physiology that accompanies leukemia development, leukemia cells acquire some properties, which are defined through the interaction with the host environment (cell-extrinsic). In particular, the immunological microenvironment acts as a fundamental background, where cell-to-cell interactions and interplay influence leukemia growth and response to chemotherapy [8].

In the clinical setting, although for many years, an anti-tumor immune response of cancer patients has been considered weakly efficient and globally anergic, the recent and successful application of a new class of compounds, such as immune checkpoint inhibitors (ICIs), which act by removing the brake on anti-tumor T cell immunity, represents the proof of principle that immune system in cancer patients is alive, and simply awaits to be reactivated. 

In this review, we will focus on current immunotherapy strategies in elderly AML. Monoclonal antibodies and bispecific T-cell engaging (BiTE) antibodies will not be discussed, as this approach will be covered in another manuscript of this Special Issue.

## 2. Immunosenescence and Immunoaging (Biological Background)

**Figure 1:**
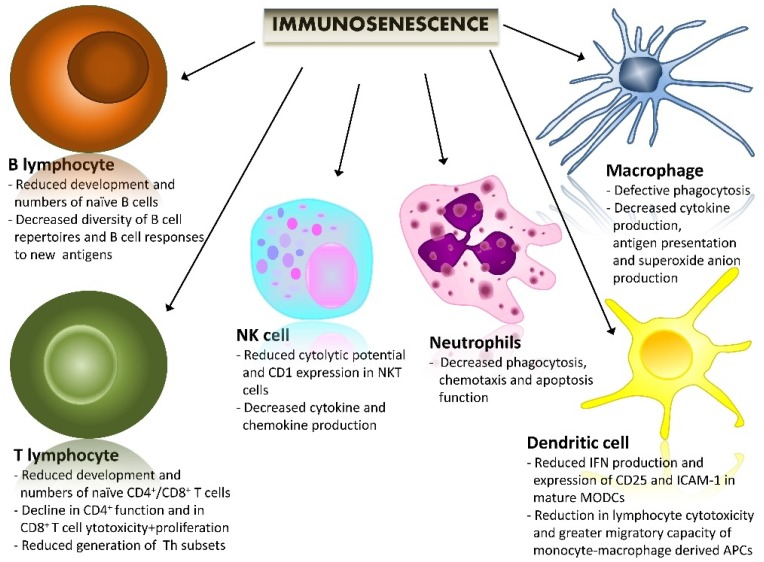
The impact of immunosenescence on an immune system’s cells. A brief summary of the most important age-related immune changes. Immunosenescence is associated with a wide variety of alterations of immune functions. Here is a brief description of these changes, subdivided into the different cell components of an innate and adaptive immune system. NK cell: natural killer cell.

Along with age increasing, the immune system undergoes a gradual process of remodeling, named immunosenescence, which alters long-term immune responses, and consequently, profoundly subverts the capacity of the host to deal with infections and other inflammatory *stimuli* (Figure 1) [9,10].

As compared to young healthy individuals, elderly subjects globally show a decline of most immune parameters, which have been correlated with their increased propensity to develop a wide range of diseases, such as infections, autoimmune disorders, chronic inflammatory diseases, and more importantly, cancer [11]. In recent years, this negative view of immunosenescence has been challenged by a series of reported studies on centenarians, where aging-related immune alterations have been demonstrated to be part of a positive adaptation of the immune system to the inflammatory microenvironment rather than a detrimental exhaustion of the reactivity of the immune system [12]. Indeed, these results may indicate that, although age-related changes in immune responses may lead to various diseases, they may also be crucial for longevity. 

Immune changes associated with aging involve an innate and adaptive immune system. Although in the elderly, the innate response has been shown to be relatively maintained, important alterations of the innate immunity have been also described [12,13]. In particular, these studies indicate that aging innate immune cells are in a state of sustained activation at the basal level, as demonstrated by an increase of homeostatic cytokine production and myeloid cell number, which is coupled with reduced cellular functions, i.e., phagocytosis, chemotaxis and free-radical production, under stress conditions. With respect to the adaptive immune system, many alterations have been described in aging [14]. In particular, aging is associated with two important changes in T cell subpopulations: (1) a decrease in naïve T cells, mainly due to combined thymic involution at puberty and hematopoietic stem cell insufficiency; and (2) an increase in primed-memory T cells and T regulatory cell number. Although the decrease in naïve T cells leads to reduced capacity to respond to neo-antigens, the increase in memory T cells may allow the adaptive immune system of the elderly to globally respond to antigenic stimulation. This is what occurs in the centenarians, whose TCR repertoire is relatively maintained and capable to respond to antigen stimulation. With regard to B cells, aging correlates with increased B-cell autoantibody production and decreased B-cell immunoglobulin production. 

With that in mind, as referred to cancer, ageing has been associated with a number of alterations of the immune system, such as the exhausted differentiating capacity of hematopoietic stem cells into lymphoid cells and the reduced function of antigen-presenting cells and of anti-tumor T cells [11]. A better understanding and characterization of these changes has important clinical implications in the immunotherapy era, when new and effective immunotherapeutics are under active investigation and, given their global reduced toxicity as compared to standard conventional therapies, e.g., chemotherapy, these changes are likely to be included in the management of elderly patients. 

## 3. Immunotherapy for Elderly Patients with AML: Different Strategies to Harness the Immune System against Leukemic Clones 

Acute myeloid leukemia is most common in the elderly population [4]. However, elderly patients are thought to be unfit for intensive treatment because of the high risk of fatal toxicity, thus requiring other therapeutic approaches to optimizing their clinical outcomes [5]. Indeed, although the CR rate in elderly AML patients fit to intensive therapies ranges between 60% and 80%, a relapse rate is still a matter of concern, thus reducing the 5-year overall survival (OS) to less than 10% [3,4,5]. A dismal clinical outcome in the elderly is not only due to the presence of more unfavorable biological features with respect to the younger population, but also owing to the presence of multiple and often severed comorbidities [5]. Thus, consolidation strategies based on allogeneic stem cell transplant (allo-SCT) are limited to a very small number of elderly patients who achieve CR. As a consequence, the persistence of a (sometimes minimal) residual disease is a major hurdle, and the post-remission management of AML patients aged >65–70 years represents an unmet medical need in these patients [5].

Advances in the immunotherapy of AML have created opportunities for improving the outcome of the elderly population. Enforcing a tumor-specific immune response through the re-direction of the adaptive immune system, which links outstanding specificity with powerful cytotoxic effector functions, has proven particularly compelling [15]. This may be coupled with immune checkpoint blockades and conventional therapies for optimal effects. Natural killer (NK) cells have shown effectiveness in this disease [16]. New methods to optimize the targeting and activation of AML cells show potential [15]. Last but not least, adoptive immunotherapy with tumor-specific T cells with T cells re-directed using genetically introduced TCR or chimeric antigen receptors, has shown promising activity, in both pre-clinical and clinical settings [16,17]. 

**Figure 2:**
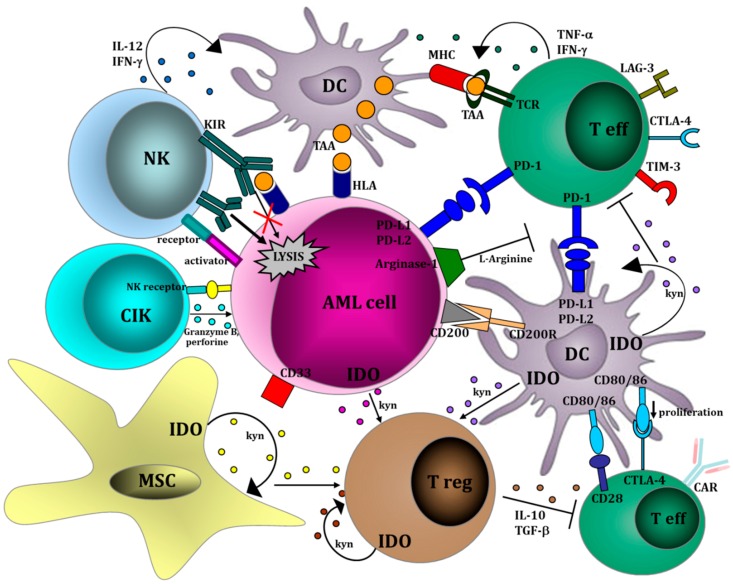
Relevant immunological pathways therapeutically targetable in AML. Within a leukemic microenvironment, AML cells interact with a variety of cells, such as T effectors, T regulatory cells, DCs, NK cells and mesenchymal stromal cells. AML is capable of creating a microenvironment, where both innate and adaptive immune responses are profoundly deregulated. The result of such a complicated cellular network is the activation of the immune response or, alternatively, the suppression of anti-leukemia immunity. The major aim of the new therapies is to harness the immune system against AML both by implementing the cytotoxic effector pathways (i.e., CTLs, NK cells and CIKs) and/or by inhibiting the tolerogenic mechanisms, (i.e., Tregs and MSCs). MSC: mesenchymal stem cell; IDO: indoleamine 2,3-dioxygenase; AML: acute myeloid leukemia; NK: natural killer cell; KIR: killer immunoglobulin receptor; CIK: cytokine-induced killer cell; DC: dendritic cell; T eff: effector T cell; Treg: regulatory T cell; kyn: kynurenine; PD-1: programmed cell death 1; PD-L1: programmed cell death ligand 1; PD-L2: programmed cell death ligand 2; Il-12: interleukin 12; IL-10: interleukin 10; IFN-g: interferon gamma; TNF-a: tumor necrosis factor alpha; TGF-b: transforming growth factor beta; MHC: major hystocompatibility complex; TAA: tumor associated antigen; HLA: human leukocyte antigen; LAG-3: lymphocyte-activation gene 3; TIM-3: T-cell immunoglobulin and mucin-domain containing-3; CTLA-4: cytotoxic T-Lymphocyte Antigen 4; CD200 R: CD 200 receptor. This figure was originally reported on the published paper [18].

The most relevant strategies to harness the immune system against AML are shown in Figure 2 and listed in Table 1.

### 3.1. Vaccines

Many leukemia-associated antigens (LAAs) have been identified in the last years [19,20,21,22]. Stimulation of autologous T cells by in vivo immunization with leukemia-associated antigens is an innovative strategy to combat relapse in AML. 

Peptide vaccines developed from LAAs, such as Wilms’ tumor 1 (WT1) antigen, Proteinase-3 (PR3) peptide, preferentially expressed antigens of melanoma (PRAME) and receptors for hyaluronic acid-mediated motility (RHAMM), have been explored and/or are under clinical investigation for the treatment of AML [19,20,21,22]. The results of these studies indicate the feasibility of vaccinating AML patients against these antigens. In most cases, immunological responses are observed. Even if these studies support the proof of principle that antigen-specific immune responses may be elicited in AML patients through vaccination, the clinical results are globally unsatisfactory, with few significant clinical responses. 

Dendritic cell (DC) vaccines are eminently equipped to stimulate antigen-specific T-cell immunity in view of their role as the most potent antigen-presenting cells of the immune system. This explains the strong interest in the use of these cells for cancer vaccination strategies. There are currently two categories of DC-based vaccines. The first is DC vaccines, which are prepared in vitro by using autologous normal DCs, generated from leukemia patients in CR loaded with tumor antigens. The reinfusion of these DCs can then induce anti-tumor immunity. The second category is transfected-DC based vaccines. Among the co-stimulatory machinery delivered to DCs, several clinical trials evaluated a multiplicity of approaches to loading LAAs onto DCs, comprising peptides, proteins, DNA/RNA-encoding tumor associated antigens, or whole tumor cells.

Recently, 30 patients with AML in remission but at very high risk of relapse were vaccinated with autologous DCs loaded with the WT1 antigen by means of messenger RNA (mRNA) electroporation [23]. Fifteen patients aged more than 65 years received the vaccine. The median overall survival in the elderly population was 17.9 months, and the OS benefit was linked to the induction of WT1-specific CD8^+^ T-cell immunity [23]. Unfortunately, results in the elderly population were significantly worse than those in the whole population, suggesting that immunosenescence may reduce the probability of responding to therapies aimed to boost T-cell immunity.

### 3.2. Natural Killer Cell Therapy

NK cells are potent effectors, which can target and kill leukemia cells without prior exposure to those cells [24]. NK cells recognize their targets, including tumor cells, in a major histocompatibility complex (MHC)- and antigen-independent manner, in a different way from T cells. NK cells are circulating CD3^−^ lymphocytes, which express CD56 or CD16 and inhibitory receptors called killer-immunoglobulin receptors (KIRs). It is well known that NK cells have a major role in the eradication of residual AML cells after haploidentical SCT [25], boosting graft-versus-leukemia (GVL) effects without exacerbating graft-versus-host disease (GVHD). Nonetheless, adoptive immunotherapy with haploidentical KIR-mismatched NK cells, administered to high-risk AML patients as consolidation therapy, was proven to be effective for the reduction of the relapse rate. Several groups, including our group [26,27], reported the feasibility of selecting and infusing highly purified, T-cell-depleted and KIR-mismatched NK cells to consolidate remission in high-risk patients with AML.

Although these results suggest that treatment with haploidentical and KIR-mismatched NK cells is a safe and potentially valuable approach to reducing the risk of relapse in patients with AML, several criticisms still remain, and additional clinical trials are required. 

One open issue is if the enhancement of NK cell activity will be required to provide optimal antileukemic effects. Potential methods to increase NK cell numbers and activity include the expansion of activated NK cells [28] and the addition of RXRγ agonists or lineage-specific antibodies, such as anti-CD33 [29,30]. Another method to enhance NK activity is the use of anti-KIR antibodies to block inhibitory KIRs; this approach was recently shown to be safe in patients with AML [31]. The depletion of host regulatory T cells (Tregs), which may inhibit the proliferation of donor NK cells, may also improve the efficacy of NK cell therapy. A recent clinical trial demonstrated that depletion of host Tregs by an IL-2 diphtheria toxin fusion protein was associated with increased NK cell expansion and higher response rates in adults with relapsed AML [17,32].

Within clinical trials, biology-driven approaches have the potential to identify an array of biomarkers, which may be used to predict clinical responses to NK immunotherapy and/or to guide clinical decision processes. Some of these biomarkers may derive from donor repertoire, whereas some others are related to host modifications after NK cell infusion. The KIR-L mismatch between a recipient and a donor could have a predictive value in terms of clinical response. On the donor side, based on the results from the setting of HLA-matched SCT transplant [25], specific HLA/KIR subtype combinations may allow to select the optimal NK cell donor, thus optimizing the potential benefit of KIR–KIR-L mismatch between the donor and the recipient [16]. On the other side of the recipient, different components of leukemic immunological microenvironment, such as Tregs [17] and mesenchymal stromal cells (MSCs) [16], have been correlated with reduced activity of NK cells, being optimal candidates to become predictive biomarkers of response to NK-cell based adoptive immunotherapy. Although these biological factors have been poorly investigated at the clinical level, future immunotherapy trials are expected to take into accounts some of these parameters, which may be correlated with response to NK immunotherapy.

## 4. Chimeric Antigen Receptor (CAR) T Cells

To overcome tolerance to tumors that results from deficiencies in the T cell receptor repertoire, T cells are genetically modified to express CARs for a specific cell-surface antigen. CARs are engineered cell surface molecules that link a target-cell ligand-recognition domain to signaling regions from the TCR. CARs are synthetic molecules resulting from the fusion of an extracellular antigen-binding domain and intracellular signaling domains, capable of activating T cells. Of foremost importance in CAR design is the recognition domain. AML comprises a diverse array of myeloid cancers, and it is unlikely that a single target will be effective for all subtypes. As a fact, the choice of a specific cell-surface antigen in AML is more demanding, as classic LAAs are also frequently expressed in the normal myeloid cell compartment [33]. 

Possible antigens to target with CARs are:

(1) CD123, which is over-expressed in AML compared with normal hematopoietic cells [34,35]. CD123 expression is mainly restricted to cells of the myeloid lineage, is absent in T cells and shows limited expression on hematopoietic stem cells. Recently, a myeloablative CAR-based therapy targeting CD123 (CART123) has shown, in a mouse model, a potent effector activity against cell-line and primary AML [36]. Moreover, CART123 led to long-term survival of mice engrafted, and resulted in the establishment of a T-cell memory pool able to reject diseases [36]. Mardiros et al. [35] developed and evaluated 2 CARs containing a CD123-specific single-chain variable fragment, in combination with a CD28 costimulatory domain and CD3ζ-signaling domain, targeting different epitopes on CD123. These second generation CD123 CAR T cell activated T-cell effector functions against poor-risk primary AML patient samples. Additionally, T cells obtained from patients with active AML and genetically modified to express CAR 123 were able to lyse autologous AML blasts in vitro. Finally, a single injection of CD123 CAR T cells exhibited a significant antileukemic activity in vivo against a xenogeneic model of disseminated AML [35]. Currently, a trial with anti-CD123 CARs for relapsed or refractory AML patients was recently started and is actively recruiting patients (NCT02159495).
(2) CD33. This differentiation antigen is predominantly expressed on myeloid cells, and it is also expressed in a subset of T cells, making it a non-ideal target for a CAR based therapy. A Chinese phase I clinical trial has studied the feasibility of anti-CD33 CAR in the treatment of relapsed or refractory AML. Only one patient was treated. Suggestions of a beneficial effect were present, but severe side effects, such as fever, cytokine release syndrome and pancytopenia, were reported [37].
(3) LeY dicofusylated carbohydrate antigen). Recently, a small study tested the feasibility and the safety of CAR anti-LeY (therapy in patients with relapsed AML, in whom the blasts were shown to express LeY). The transducted and expanded autologous CAR T cells were successfully and safely infused in 4 patients with high-risk AML, showing tissue specific localization, long-term persistence and antileukemic efficacy [38].

Beyond identifying an optimal target antigen, many obstacles will be expected with CAR-modified T cells in AML, especially in the elderly population. Among these, the effects of PD-L1, IDO and other inhibitory molecules on disablement of the therapeutic response, and localization and persistence of adoptively transferred therapeutic T cells with AML blasts will probably represent the major obstacles for the success of CARs in AML.

## 5. Checkpoint Inhibitors

Leukemic cells may evade immune recognition and killing by cytotoxic T lymphocytes through the inhibition of intrinsic immune responses by a multiplicity of mechanisms, such as secretion of immunosuppressive cytokines, aberrant antigen expression by leukemia cells, expression of inhibitory enzymes in the tumor microenvironment and activation of immune checkpoint pathways [39]. All these mechanisms usually lead to T cell dysfunction and/or exhaustion. However, there is a large confusion, in the field of aging, with respect to the number and distinction between senescent T cells, which may be functionally inert and exhausted cells which may be functionally “dormant”. [40,41]. This distinction is crucial when immune functions in relationship to aging are considered. Exhausted T cells can be reawakened through the modulation of some surface receptors, namely the ICIs, and then resume their functions [41,42,43,44]. Of these receptors, the most important ones are PD-1, CTLA-4, LAG-3 and TIM-3. 

Blockage of immune checkpoints has emerged as a highly promising approach to augment innate anti-tumor immunity to treat malignancy. In particular, two co-receptors, CTLA-4 and PD-1, provide crucial inhibitory signals that down-regulate T-cell functions in the context of antigen recognition [42,43,44]. CTLA-4 (cytotoxic T-lymphocyte associated antigen 4, CD152) is an inhibitory receptor predominantly expressed on activated T cells and on Tregs that suppresses activation of effector T cells by competing with CD28, a co-stimulatory molecule on T cells, for binding to ligands CD80 and CD86. Several preclinical and clinical trials have reported that the CTLA-4 blockade with Ipilimumab can establish an anti-leukemic effect without GVHD after allogeneic HSCT and restore anti-tumor reactivity for patients with relapse. Although durable responses were observed, the efficacy of CTLA-4 inhibition needed to be confirmed. Programmed death-1 (PD-1, CD279) is another inhibitory receptor that belongs to the B7/CD28 family. PD-1 was identified on T cells undergoing apoptosis and its major role in inhibiting T cell activation and effector functions in an inflammatory environmental and controlling autoimmunity in peripheral tissues [45]. 

A few years ago, the PD-1/PD-L1 pathway was shown to be involved in immune escape in a murine model of AML, thus resulting in AML progression [7]. Starting from this rationale, Yang et al. demonstrated that PD-1, PD-L1, PD-L2 and CTLA4, are aberrantly upregulated in 8–34% of CD34+ AML cells [46]. Patients with lower expression of PD-L1 showed a trend towards better survival; that, however, was not statistically significant (31.5 months versus 16.2, *p* = 0.24) [46]. Moreover, PD-L1 expression correlated with progression from MDS overt AML, and PD-L1, PDL2, PD-1 and CTLA4 was induced by treatment with hypomethylating agents (HMA) in a concentration dependent manner [46]. Exposure to decitabine resulted in demethylation of PD-L1 in AML cell lines, and the demethylation effect was also observed in HMAs treated MDS and AML patients [46].

The association between the demethylation of the PD-1 promoter during HMAs treatment in MDS/AML patients and a worse outcome has been independently reported by another group [47]. The overall response rate was significantly lower (8% vs. 60%, *p* = 0.014), and a trend towards a shorter overall survival (*p* = 0.11) was observed in MDS/AML patients, in whom the demethylation of the PD-1 promoter occurred [48]. This is probably due to the fact that the expression of PD-1 on activated T cells is regulated by DNA methylation [49], and PD-1 promoter demethylation correlates with an increase in PD-1 expression [49]. Therefore, the activation of the PD-1 checkpoint during HMA treatment can be a possible resistance mechanism to these drugs, widely used in elderly AML patients. These findings have resulted in multiple clinical trials combining HMAs with immune checkpoint blockades. A number of trials combining HMAs with PD-1/PD-L1-based therapies have recently started enrollment for AML and MDS, including azacitidine with the anti-PD-1 antibody nivolumab (NCT02397720), azacitidine with or without the anti-PD-L1 antibody durvalumab (NCT02775903) and azacitidine with or without the anti-PD-L1 antibody atezolizumab (NCT02508870) [50]. Notably, among these studies, Daver et al. [51] are evaluating the association of nivolumab (Opdivo, BMS-936558, Bristol-Myers Squibb, New York, NY, USA) and azacitidine in a phase I/II trial in patients with relapsed/refractory AML and in frontline therapy for older patients with AML (≥65 years) who are not fit for intensive chemotherapy (NCT02397720) [51]. Up to now, 53 patients with a median age of 9 years were enrolled. The nivolumab/azacitidine combo preliminarily showed significantly an improved overall response rate, and a median PFS as compared to a historical control of patients treated with 5-azacitidine alone at the same institution, with a reduction in 8-week mortality. Notably, the response rates were higher in patients with a diploid karyotype. Two interesting biological observation were reported: (1) a higher total CD3 and higher CD8^+^ T cell infiltrate was observed in pre-therapy BM aspirate of patients achieving CR/CRi; and (2) responders had progressive increase in BM CD8^+^ and CD4^+^ T cell infiltrate [50,51].

Starting from this background, larger clinical trials are currently ongoing in order to determine the role of checkpoint inhibition, with or without hypomethylating agents in myeloid malignancies, not only in the elderly population (NCT02397720, NCT 02530463, NCT02464657, NCT01822509) [50]. Moreover, dual combination of nivolumab (PD-1) and ipilimumab (CTLA-4) with azacitidine in relapsed and in frontline elderly AML therapy has recently begun enrollment (NCT02397720).

## 6. How Immunosenescence May Influence the Different Immunotherapy Strategies in AML Patients

The impressing clinical results obtained in advanced cancer patients with the use of a variety of strategies harnessing the host immune system has definitively demonstrated that in cancer, including leukemia patients, the anti-tumor immune response is hampered, but is not completely abrogated. In particular, the efficacy of ICIs, which remove the brake from the anti-tumor immune response, clearly supports the notion that the T cell repertoire of cancer and leukemia patients can be restored and potently redirected against tumor cells. In this context, it is intriguing to ask whether senescent immune cells from cancer and leukemia patients may be constitutively different in responding to immunotherapies. In the field of solid tumors, such as melanoma and non-small cell lung cancer, where ICIs have proven to be highly effective, the impact of immunosenescence on the effectiveness of these drugs has been recently evaluated [52]. The first clinical results suggest that increasing age is a parameter affecting the response to ICIs. In particular, the efficacy of PD-1 inhibitors in lung-cancer patients has proven to be negatively affected by immunosenescence [53]. These pathways are emerging as relevant in the hematological field. In particular, PD-L1 expression was shown to be also present on AML cells [7,54,55] and was correlated with AML progression, independently from other biological prognostic factors. In line with the results reported for solid tumors, experiments conducted in a murine model of AML indicate that the PD-1/PD-L1 pathway promotes immune escape, thus resulting in AML progression. These data support a rationale for clinical trials examining the effect of ICIs in AML patients, which are currently ongoing. In these studies, the impact of increasing age is not specifically addressed. However, it should be noted that a different composition in the elderly as compared to young patients of T-cell repertoire with regard to the expression of inhibitory receptors, as well as skewing of T-cell subset toward Tregs and/or exhausted T cells, may significantly impact on the clinical response to these new drugs. Under this viewpoint, we propose that the underlying background of immunosenescence should be specifically addressed in future clinical trials, where novel immunotherapies are used [56,57]. A better analysis of age-related immune changes and their impact on biological and clinical responses to new drugs and strategies, involving patients’ immune systems, is likely to provide a new set of age-related biomarkers to be correlated with immunological and clinical parameters of response. This approach is in line with the emerging notion that patient’s immune background, including the different composition of immunological leukemia microenvironments, plays a critical role for the development of AML and may crucially affect the response to therapeutical interventions [56,57]. In this scenario, immunosenescence is certainly a prominent issue. 

## 7. Conclusions

It is well known that the clinical management of elderly AML patients represents a major task for hematologists. Many and, in some cases, overwhelming factors greatly impact on the effectiveness of different therapeutic strategies on the outcome of these patients, which still remains poor. In this scenario, it is time to ask whether a prominent, if not exclusive, and clinical approach to the choice of which therapy for which patient is still acceptable. Indeed, senescence is associated with a physiological alteration of a wide variety of biological functions, which is likely to have an impact when a specific drug is used in the elderly as compared to young patients. With that in mind, in the era of immunotherapy, we consider immunosenescence as the optimal background to start investigating a biology-driven approach to AML therapy in elderly AML. By taking into account the physiological age-related changes of immune response, more personalized and tailored use of the new drugs and strategies harnessing the immune system against AML has the potential to increase their efficacy and impact on clinical outcomes.

## Figures and Tables

**Table 1:** Strategies to harness an immune system against AML.

Pathway		Therapeutical Action	Effect
Type	Mechanism		
Checkpoint inhibitors	PD-1/PD-L1	- mAb anti-PD-1 - mAb anti-PD-L1	- Increased T-cell cytotoxicity - Increased DC function as APCs
	KIR	- mAb anti-KIR	- AML cell lysis
Tolerogenic molecules	Arginine	- human recombinant arginase	- Prevention of immune tolerance
	IDO	- IDO1-inhibitor	- Prevention of immune tolerance
Adoptive cell-therapy	NK cells	- adoptive cell therapy	- AML cell lysis
	CAR-T cells	- adoptive cell therapy	- AML cell lysis
	TCR-edited T cells	- adoptive cell therapy	- AML cell lysis
Antigens/Dendritic cells loaded with antigens	WT1, RHAMM, PR-3, DC/WT1	- vaccines	- Specific AML cell lysis
